# An empirical study on the psychological impact of medical AI on patients undergoing dental surgery

**DOI:** 10.1038/s41598-025-29754-0

**Published:** 2025-12-03

**Authors:** Na Zhu, Jianing Bian, Zixuan Dong, Na Zhu

**Affiliations:** Affiliated Hospital of Chifeng University, No. 48 Wangfu Street, Chifeng City, 024000 Inner Mongolia China

**Keywords:** Psychological experience, Dental surgery, Patient perception, Psychology, Medical research

## Abstract

With the rapid integration of artificial intelligence technology into the medical field, this study focuses on dental surgery and systematically examines the complex impact mechanisms of AI on patients’ treatment anxiety and postoperative satisfaction through three progressive experiments involving a total of 470 participants. Guided by a TAM-informed, trust-augmented framework, we examine the mediating role of trust in AI and the moderating effects of gender and information transparency. Key findings indicate: (1) AI technology significantly reduces patient treatment anxiety levels (*p* < 0.001) and enhances postoperative satisfaction (*p* < 0.001); (2) Across the three studies, trust in AI functioned as a partial mediator overall. The mediation did not emerge in the text-based simulation (Study 1), emerged in the video simulation (Study 2), and became robust in the clinical field study (Study 3; indirect effect 95% CI [− 0.89, − 0.21]) while the direct paths remained significant; (3) Gender differences significantly moderate the effects of AI technology, with female patients showing lower levels of technology trust; (4) Technology transparency positively moderates the therapeutic effect of AI application. Under high transparency conditions, the anxiety relief effect of AI technology (ΔM = 3.04) is significantly stronger than that under low transparency conditions (ΔM = 2.06). Collectively, the findings indicate that trust in AI operates as a partial, context-dependent mediator whose magnitude increases with ecological validity and transparency, clarifying when and how AI use relates to patient anxiety and satisfaction.

**Supplementary Information:**

The online version contains supplementary material available at 10.1038/s41598-025-29754-0.

## Introduction

In recent years, the application of artificial intelligence (AI) in healthcare has expanded significantly. In particular, AI-assisted systems have emerged as important tools in dental surgery, owing to their precision and operational stability. Compared with traditional procedures, AI-assisted surgeries demonstrate notable advantages, including reducing medical errors, enhancing surgical accuracy, and shortening recovery times^1,2^. However, existing research primarily focuses on the clinical outcomes of AI technology, with limited attention to its impact on patients’ psychological experiences, particularly treatment-related anxiety and postoperative satisfaction. Given that dental surgery inherently tends to provoke anxiety, the introduction of AI as a “non-human” operator may further intensify this psychological burden^3^.

Previous studies have shown that patients’ treatment-related anxiety not only affects their overall medical experience but may also interfere with treatment outcomes. In traditional surgical settings, effective doctor–patient communication is considered a key factor in alleviating patient anxiety^4^. However, the integration of AI technology alters the traditional interaction pattern, potentially causing patients to experience a sense of insecurity due to the absence of a conventional doctor–patient relationship. While the classic Technology Acceptance Model emphasizes Perceived Usefulness and Perceived Ease of Use leading to Behavioral Intention and eventual Use, our study does not attempt to test that full structure. Instead, we adopt a TAM-informed perspective and focus on trust in AI as a proximal determinant of psychological outcomes, namely anxiety and satisfaction, in clinical settings. Therefore, fostering patients’ trust in AI may serve as a critical pathway for optimizing treatment experiences in AI-assisted surgical procedures^5^.

Notably, patients’ acceptance of AI technology can be influenced by multiple factors. One significant dimension is gender, as previous studies have shown that female patients generally exhibit higher levels of medical anxiety and lower levels of technology acceptance^6^. Additionally, information transparency during the surgical process may influence patients’ attitudes toward AI technology. When patients receive timely and clear updates about the progress of AI-assisted procedures, their level of trust in the technology is likely to increase significantly^7^. However, systematic empirical research examining the combined effects of these factors on patients’ psychological responses remains limited.

This study investigates the impact mechanism of AI-assisted dental surgery on patients’ treatment anxiety and satisfaction through three progressive experiments. It examines the mediating role of technology trust and explores the moderating effects of gender differences and technology transparency. These findings not only advance the application of technology acceptance theory in medical contexts but also offer practical guidance for optimizing the clinical implementation of AI-assisted surgeries. In particular, by elucidating the role of technology transparency, this research provides valuable insights into enhancing patients’ acceptance of AI technology and improving their overall treatment experience.

## Literature review and research hypotheses

### AI technology usage, treatment anxiety, and post-operative satisfaction

#### AI technology usage and treatment anxiety

The application of AI in the medical field not only enhances surgical efficiency and precision but also potentially influences patients’ psychological experiences, particularly their levels of treatment-related anxiety^8^. Previous research indicates that unfamiliarity and uncertainty are major sources of patient anxiety. Patients undergoing robot-assisted surgery for the first time often exhibit heightened preoperative anxiety ^9^,primarily due to concerns about the safety and reliability of AI technology, as well as an instinctive resistance to non-human operators^10^.

However, as AI technology continues to mature, its potential in reducing patients anxiety is gradually emerging^11^. Researchers have found through comparative analysis that patients undergoing AI-assisted surgery exhibit lower physiological stress responses compared to those receiving traditional surgery, suggesting that AI technology may offer advantages in stabilizing patient emotions^12^.Further psychophysiological research demonstrates that during standardized AI-assisted procedures, patients exhibit significantly lower heart rate variability, cortisol levels, and other stress indicators compared to those undergoing traditional surgery^13,14^. These findings provide objective evidence for AI technology’s improvement of patients’ psychological experiences.

Moreover, the impact of AI technology on patient anxiety may depend on the specific application method^15^. When AI systems can display surgical progress in real-time and provide precise operational data, patients’ technology trust significantly increases, and anxiety levels correspondingly decrease^16,17^. This suggests that AI technology can reduce patient anxiety not only by improving surgical precision but also by enhancing the controllability and predictability of the surgical process^18,19^.

Based on this evidence, it can be inferred that AI technology, by enhancing surgical controllability and predictability, may offer significant advantages in reducing patient anxiety^20^. Therefore, this study proposes the following hypothesis:

##### H1a

Higher levels of AI technology use are associated with lower levels of patient treatment anxiety.

#### AI technology usage and post-operative satisfaction

Postoperative satisfaction represents a patient’s overall evaluation of the treatment experience, primarily influenced by treatment efficacy, the surgical process, and the quality of doctor–patient communication^21–23^. With the deepening application of AI technology in the medical field, its impact on patient satisfaction has increasingly attracted research attention. Enhanced surgical precision can substantially influence patient satisfaction. By reducing human error and ensuring consistent operation, AI technology significantly improves surgical outcomes, thereby directly contributing to higher patient satisfaction^24^.However, the impact of AI technology on postoperative satisfaction may be dual-faceted. While AI enhances surgical precision, its lack of human interaction may weaken the emotional connection inherent in traditional doctor-patient relationships. This emotional deficit may partially offset the improvements in satisfaction resulting from enhanced surgical outcomes^25^.

Meanwhile, this emotional deficiency can be mitigated by optimizing the interactive design of AI systems. Surgical plans that integrate AI technology with human-centered interaction—such as enhanced explanations and guidance from doctors during surgery—help maintain both the precision of AI operations and the emotional connection between doctors and patients. Experimental results indicate that patients receiving such combined care report significantly higher satisfaction compared to those undergoing traditional surgery^26^.

From a long-term perspective, the impact of AI technology on patient satisfaction may increase in conjunction with improved recovery outcomes. Studies have shown that patients undergoing AI-assisted surgery experience lower complication rates. These benefits are especially reflected in higher postoperative satisfaction evaluations^27^.

We sincerely thank the reviewer for pointing out the conceptual distinction between “post-operative satisfaction” and “expected satisfaction.” We agree that, in Studies 1 and 2, participants only imagined the treatment scenarios rather than undergoing actual procedures. To clarify this, we have added the following explanatory sentence to Sect. “AI technology usage and post-operative satisfaction” (AI Technology Usage and Post-Operative Satisfaction):

In Studies 1 and 2, although the variable is labeled as post-operative satisfaction for consistency across studies, the outcome in these experiments actually reflects participants’ expected satisfaction following the hypothetical treatment.

This clarification acknowledges the hypothetical nature of satisfaction ratings in the first two studies and ensures terminological consistency throughout the manuscript.

Although AI technology may have limitations in doctor-patient communication, its advantages in enhancing surgical precision and accelerating recovery speed may still bring higher overall satisfaction^28^. Therefore, this study proposes the following hypothesis:

##### H1b

A higher level of AI technology utilization is associated with greater postoperative patient satisfaction.

### Mediating Effect of Technology Trust

Building on extensions that integrate trust into technology acceptance, we conceptualize trust in AI as a key proximal mechanism shaping psychological responses, specifically anxiety and satisfaction, in dental surgery contexts. This positioning links acceptance theory with outcomes that are immediately relevant to clinical practice. Building on trust-integrated extensions of technology acceptance research, we conceptualize trust in AI as a key mechanism through which the use of AI may influence patients’ psychological experiences^29^. Existing research indicates that technology trust not only directly influences patients’ acceptance of AI systems but also shapes their psychological experiences during treatment by modifying their risk perceptions and expectations^30^. Researchers emphasize that patients’ trust in technology is often established during their initial encounter with AI systems, and these early impressions persistently shape their subsequent treatment experiences^31^. Further research emphasizes that medical institutions should implement targeted interventions at critical patient–AI interaction points to foster and sustain high levels of technology trust^32^.

The mediating role of technology trust in alleviating treatment anxiety has been empirically validated by numerous studies. Technology trust influences patients’ anxiety levels primarily through three mechanisms: first, by reducing patients’ subjective risk perception of surgery; second, by enhancing their sense of control over the treatment process; and finally, by fostering more positive expectations regarding treatment outcomes^33^. Physiological monitoring has confirmed that patients with higher levels of technology trust exhibit lower stress hormone concentrations and greater stability in heart rate variability during surgery. Additionally, the research revealed that the development of technology trust is cumulative, with each positive interaction reinforcing patients’ trust and creating a virtuous cycle^34^.

Regarding postoperative satisfaction, the mediating role of technology trust is more complex. Technology trust not only directly influences patients’ subjective satisfaction but also indirectly enhances treatment outcomes by promoting treatment adherence^35^. Patients with high levels of technology trust are more likely to adhere to medical advice and actively engage in rehabilitation, behaviors that collectively enhance treatment outcomes. Moreover, a bidirectional relationship exists between technology trust and patient satisfaction: initial trust influences patients’ evaluations of the treatment process, while the progressive realization of positive treatment effects further strengthens their trust in the technology^36^. This dynamic interactive process explains why some patients’ satisfaction evaluations significantly improve over time^37^.

The mediating effect of technology trust may be moderated by multiple factors. Patients’ individual characteristics, such as age and education level, previous medical experiences, and the complexity of the current surgery all affect the formation and action mechanism of technology trust^38^. Especially in elderly patient groups, establishing technology trust often requires more time and detailed explanations. Meanwhile, the technology presentation method of medical institutions and the quality of doctor-patient communication are also key factors affecting the formation of technology trust^39^.

Based on the above analysis, we propose the following: When patients exhibit higher levels of trust in AI technology, its effectiveness in reducing anxiety becomes more pronounced. Furthermore, technological trust can strengthen the positive effect of AI on postoperative satisfaction; however, this mediating effect may partially depend on doctor–patient communication and surgical outcomes.

#### H2a

 Trust in AI partially mediates the relationship between AI use and treatment anxiety, and its magnitude increases as ecological validity rises from text to video to field.

#### H2b

 Trust in AI partially mediates the relationship between AI use and satisfaction, with stronger mediation expected in more realistic settings.

### Moderating effect of gender

Gender differences have been widely documented in the acceptance of medical technology and associated psychological responses^40^.Numerous studies have explored differences between males and females in terms of medical technology acceptance. Females tend to show higher risk aversion tendencies and lower initial trust when facing new medical technologies. This gender difference is particularly evident in the field of AI-assisted healthcare, where female patients often need more information and assurance to establish trust in AI technology^41^. Especially in scenarios involving surgical operations, females’ sensitivity to technological risks is significantly higher than males’. This gender difference may be primarily attributed to sociocultural factors, as females typically shoulder more family responsibilities and often serve as primary healthcare decision-makers, causing them to weigh potential risks more thoroughly when considering medical options^42^. Meanwhile, sociocultural factors may also reinforce this difference, as females typically shoulder more family responsibilities, causing them to weigh potential risks more when considering medical options^43^.

Gender differences are also evident in treatment anxiety. Female patients show higher levels of anxiety than male patients when facing AI surgery^44^. This difference may stem from females’ higher sensitivity to surgical risks and stronger uncertainty about non-human operators. Even in traditional surgery, females tend to exhibit stronger medical anxiety, and the introduction of AI technology may further amplify this gender difference^45^. Through in-depth interviews, researchers found that female patients are more inclined to seek emotional support and detailed explanations, while the standardized operating procedures of AI systems may not meet this need. Additionally, gender differences in healthcare experiences may contribute to these patterns, as women typically have more frequent medical encounters and may have developed different expectations regarding physician communication and emotional support during medical procedures^46^.

Gender factors play an important moderating role in the formation process of technology trust. Compared with male patients, female patients need a longer time to establish trust in AI technology. This difference may affect the effectiveness of AI technology in reducing anxiety and enhancing satisfaction^47^. Longitudinal tracking data show that female patients’ technology trust formation exhibits obvious phase characteristics: trust levels are lower in the initial phase and need to gradually increase through multiple positive experiences. Researchers emphasize that medical institutions need to pay special attention to female patients’ needs when introducing AI technology, helping them overcome initial trust barriers through enhanced technology demonstrations and increased interactive experiences^48^.

Based on this evidence, we propose that gender influences the relationship between AI usage and technology trust, with women forming trust more slowly than men. Additionally, gender moderates the effects of AI usage on treatment anxiety and postoperative satisfaction, such that women experience less anxiety reduction and potentially smaller improvements in satisfaction.

#### H3a

 Gender moderates the relationship between AI usage and technology trust, with female patients forming trust more slowly and exhibiting greater variability than male patients.

#### H3b

 Gender moderates the relationship between AI usage and treatment anxiety, with female patients experiencing a smaller reduction in anxiety compared to male patients.

#### H3c

Gender moderates the relationship between AI usage and postoperative satisfaction, with female patients demonstrating a smaller increase in satisfaction than male patients.

### Moderating effect of technology transparency

Technology transparency, as a key environmental factor in medical AI applications, has received widespread attention from academia in recent years^49^. Technology transparency reflects the degree to which patients understand the working principles of AI systems, surgical progress, and risk control information. Research indicates that high technology transparency can significantly enhance patients’ understanding and acceptance of AI systems^50^. This transparency effect is particularly evident in complex surgeries, as patients often have stronger information needs for high-risk surgeries^51^.

The impact mechanism of technology transparency on patients’ psychological responses has been confirmed by multiple studies. Experimental research found that patients under high transparency conditions exhibit lower physiological stress responses and subjective anxiety levels^52^. Specifically, when the operating room is equipped with real-time displays showing the AI system’s operational parameters and surgical progress, patients’ average heart rates and cortisol levels are significantly lower than those in routine condition groups. Researchers believe this effect stems from the enhanced sense of control brought by information transparency^53^.

In terms of long-term effects, the impact of technology transparency on patient satisfaction is more profound. Patients in high transparency groups not only show higher satisfaction post-surgery, but their improvement in technology acceptance is also more stable. Especially in the handling process of postoperative complications, these patients show stronger understanding and willingness to cooperate^54^. Researchers emphasize that the role of technology transparency is not limited to the surgical stage but should permeate the entire treatment cycle. Continuous information feedback and progress explanations can significantly enhance patients’ confidence in the treatment plan, an effect that is particularly important in cases requiring long-term follow-up^55^.Based on the above research evidence, this study proposes three hypotheses regarding the moderating effect of technology transparency.

#### H4a

Technology transparency positively moderates the relationship between AI usage and technology trust, such that the relationship is stronger under high transparency conditions.

#### H4b

Technology transparency amplifies the anxiety-reducing effect of AI technology, with this moderating effect being more pronounced in complex surgical procedures.

#### H4c

Technology transparency positively moderates the impact of AI usage on postoperative satisfaction, with a stronger effect observed under high transparency conditions.

## Research design

All experimental procedures were approved by the ethics committee of the first author’s institution (approval numbers: fsyy2024022 and fsyy2025047).

To explore the impact mechanism of AI technology in dental surgery on patient treatment anxiety and post-operative satisfaction, this study designed three progressive experiments. Experiment 1 examined the main effect of AI usage through textual scenario simulation; Experiment 2 explored the moderating role of gender differences using video scenarios; and Experiment 3 tested the moderating effect of technological transparency in a real medical environment. This multi-level experimental design not only gradually verifies the research hypotheses but also enhances the robustness of conclusions through complementary experimental scenarios. Meanwhile, the study selected different types of subject groups (laboratory-recruited volunteers, clinical patient samples, and actual medical patients), adopted diversified experimental stimulus materials (textual descriptions, video simulations, and actual surgeries), and comprehensive measurement indicators (combining subjective evaluations and objective indicators) to ensure the internal and external validity of the research results. This experimental design strategy not only helps reveal the internal mechanism of AI technology’s impact on patients’ psychological responses but also provides reliable methodological references for subsequent research and practical applications.

**Fig. 1:**
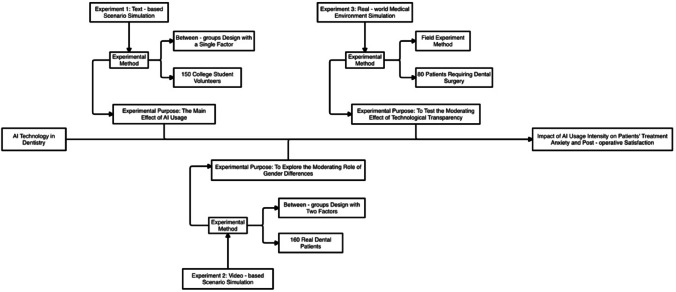
Research and design frame diagram.

This research was conducted in accordance with relevant ethical guidelines. All experimental protocols have been approved by the ethics committee of the institution where they are located. All participants have signed the informed consent form. The experimental design of this study is shown in Fig. 1:

### Experiment 1: laboratory study in textual scenarios

Experiment 1 adopted a single-factor between-subjects design, aiming to explore the direct impact of AI usage intensity (high vs. low) on patients’ treatment anxiety and post-operative satisfaction through textual scenario simulation, and to preliminarily examine the mediating role of technology trust. The experiment was conducted in an actual hospital setting, where participants not only read the scenario descriptions but also viewed real dental surgery equipment and procedural videos to closely simulate an authentic surgical environment. However, it should be noted that Experiment 1 served as a pilot study, and due to the limitations inherent in scenario-based simulations, participants’ perceived level of technological trust may have been lower than in a real surgical context.

The sample size was determined based on practical feasibility and aligns with typical ranges in exploratory psychological experiments. Thus, Studies 1 and 2 should be considered preliminary. Experiment 1 recruited 150 university students (aged 18–26) as volunteers, randomly assigning participants to high AI group (n = 75) and low AI group (n = 75). The experiment was conducted in the simulated surgical training area of a partner hospital to ensure that participants, while reading the scenario materials, could directly observe AI surgical equipment and conventional instruments and receive a brief explanation.

While acknowledging the limitations of using student volunteers rather than actual patients, this experimental design was deliberately chosen to control for confounding variables. The student sample exhibited homogeneous characteristics in terms of having no prior dental surgery experience and being at an initial stage of AI technology cognition, which allowed us to isolate the core variables of interest while minimizing the interference of previous surgical trauma memories or long-term medical anxiety.

To maximize ecological validity despite using a non-patient sample, the experiment was conducted in a carefully designed environment that replicated actual clinical conditions. The simulated surgical training area was equipped with standard dental surgical equipment, including AI-assisted surgical platforms, dental treatment units, and sterilization protocol indicators. Throughout the experiment, participants were exposed to real-time demonstrations of AI surgical equipment functionality, complemented by video recordings of actual dental procedures showing both AI-assisted and conventional approaches. This comprehensive simulation approach, while not fully replicating the psychological state of actual patients, provided participants with a highly immersive experience that helped bridge the gap between laboratory and clinical settings. The environment was specifically designed to enable participants to form meaningful perceptions of the technology while maintaining the controlled conditions necessary for initial hypothesis testing. This design strategy not only enhanced the ecological validity of our preliminary experiment but also established a methodological foundation for the subsequent studies with real patient samples.

**Fig. 2:**
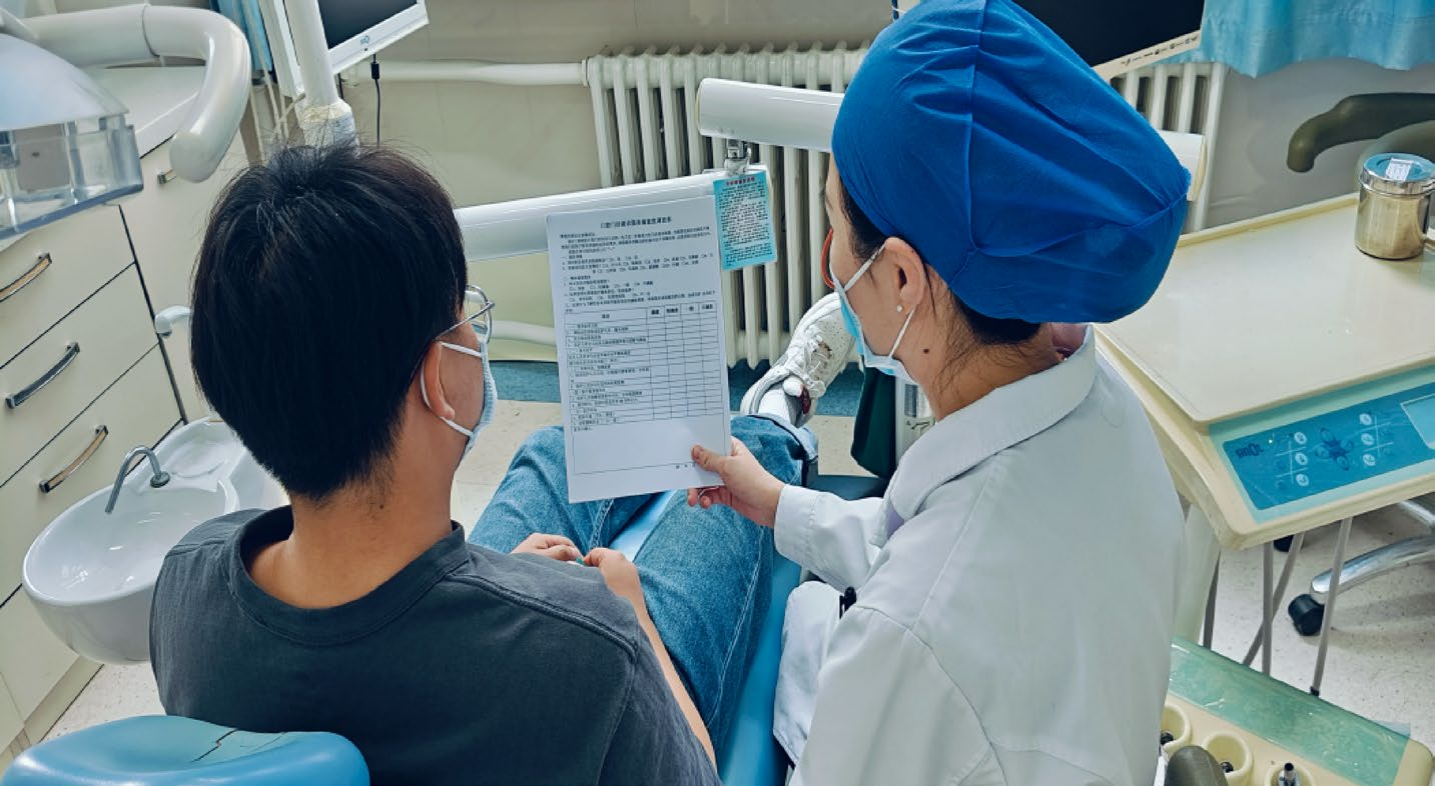
Data collection process of experiment 1.

The experiment simulated dental surgery scenarios through textual descriptions: high AI group subjects read scenario materials of “operation fully performed by AI robot, doctor only supervises,” while low AI group subjects read scenario materials of “traditional manual operation, AI only provides auxiliary advice,” to ensure subjects clearly understood the between-group differences. Each experimental volunteer receives dental health-related gifts as their rewards at the end of the experiment.The data collection process of experiment 1 is shown in Fig. 2.

Treatment anxiety was measured using the Modified Dental Anxiety Scale (MDAS)^56,57^, which includes 5 items, with Cronbach’s α = 0.66.While this reliability level meets the minimum threshold for exploratory research^58^, we acknowledge its limitations and conduct additional item analysis to ensure the robustness of our findings. The results remained consistent across different analytical approaches, suggesting the stability of our findings despite the moderate reliability.

Post-operative satisfaction was assessed using a 7-point Likert scale, with adapted questions more in line with the theme of this paper^59,60^, covering 5 items with Cronbach’s α = 0.85. Technology trust was measured using a scale adapted from the Technology Acceptance Model (TAM)^61,62^, including 5 items (such as “I believe AI technology is more precise in operation”), with a total score range of 5–35 points (Cronbach’s α = 0.81). Variables such as gender, age, oral health status, and pain sensitivity were controlled.

The main effect was analyzed through independent samples t-tests for between-group differences (high AI group vs. low AI group). The mediating effect of technology trust was tested using the Bootstrap method, with 5000 resampling calculations for 95% confidence intervals. Control variables were corrected through multivariate linear regression models to eliminate interference from confounding factors.

### Experiment 2: questionnaire experiment in video scenarios

Experiment 2 adopted a two-factor between-subjects design, aiming to validate the action mechanism of AI usage intensity (high vs. low) on patients’ treatment anxiety and post-operative satisfaction in real clinical scenarios, and to further examine the moderating effects of gender factors (male vs. female) and technology trust. The study recruited 160 real dental patients (aged 18–65, half male and half female), randomly assigned to AI group (n = 80) and traditional group (n = 80), with each experimental volunteer receiving dental health-related gifts as rewards at the end of the experiment. Participants in the AI group watched video simulations of surgical processes led by AI robots, while those in the traditional group viewed video simulations of manual operations. Both groups were exposed to standardized video simulations of surgical environments (such as mechanical arm operation details, doctor and AI collaboration processes) to enhance the experimental ecological validity without actual surgical procedures performed.

**Fig. 3:**
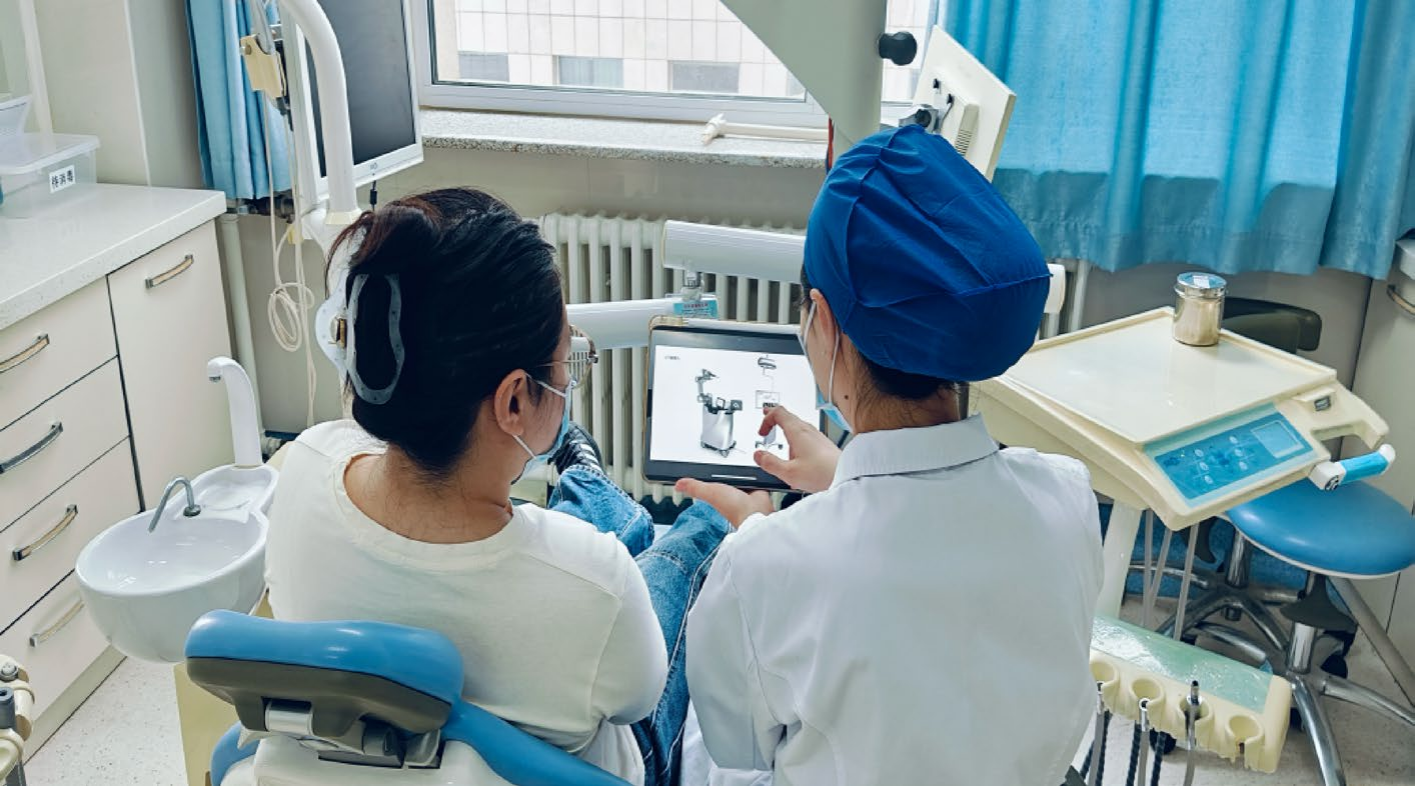
Data collection process of experiment 2.

The experiment strengthened the perception of technology trust through video manipulation: AI group videos highlighted AI technology precision (such as real-time positioning error ≤ 0.1 mm), operational stability, and risk warning functions; traditional group videos only presented doctors’ manual operation processes. Post-surgery, treatment outcome feedback was simulated through virtual interfaces (such as visualization of postoperative wounds), ensuring subjects’ immersive experience of the treatment process. Gender, as a moderating variable, controlled male–female ratio through stratified sampling and analyzed its differentiated impact on the relationship between technology trust and psychological outcomes.The data collection process of experiment 2 was shown in Fig. 3.

Experiment 2 also used the same Modified Dental Anxiety Scale (MDAS, Cronbach’s α = 0.82) and post-operative satisfaction scale (Cronbach’s α = 0.88) as Experiment 1 to measure dependent variables, while the technology trust scale (Cronbach’s α = 0.79) included dimensions such as “technological reliability” and “risk controllability”. Control variables covered age, education level, previous treatment experience, and pain sensitivity, corrected through multivariate regression models for potential confounding. Data analysis used two-factor analysis of variance (AI usage intensity × gender) to test interaction effects and validated the mediating pathway of technology trust and the moderating role of gender based on the Bootstrap method.

Through optimization with clinical patient samples and video simulation, Experiment 2 compensated for the limitation of insufficient ecological validity in Experiment 1, focusing on revealing how gender differences moderate patients’ psychological responses to AI technology. The research is expected to provide empirical evidence for the personalized application of medical AI and promote the theoretical deepening of the Technology Acceptance Model (TAM) in clinical scenarios.

### Experiment 3: field experiment in real environment

Based on findings from the first two experiments, Experiment 3 adopted a field experiment method, aiming to test the impact of AI usage intensity and technology transparency on patients’ treatment anxiety and satisfaction in a real medical environment. This experiment was conducted in the dental department of a tertiary hospital, recruiting 160 patients requiring dental surgery to participate in the study, with each patient receiving a dental health-related gift reward upon completion. Patients ranged in age from 18–65 years (data from patients over 65 was discarded as they were found in the experiment to have difficulty understanding questionnaire questions well) and were randomly assigned to four experimental groups with 40 participants in each group.

All patients enrolled in Experiment 3 were informed of the random assignment procedure and confirmed that they had no strong preference for either surgical modality before inclusion. Patients expressing explicit preferences for AI-assisted or traditional surgery were excluded prior to randomization. Random allocation was then implemented using a computer-generated list, with concealment ensured through sealed envelopes prepared by an independent coordinator. All surgeries followed the same protocol under standardized local anesthesia, without sedation, and identical postoperative care. Preoperative anxiety was assessed after consent and before the briefing, and satisfaction was measured immediately after the procedure. Clinician–patient communication was guided by a unified script to maintain consistency between groups.

The experiment adopted a 2 (AI usage intensity: high vs. low) × 2 (technology transparency: high vs. low) between-subjects design. “AI usage intensity” was manipulated through the degree of AI robot participation in the surgical process: high AI group was led by AI robots with doctors providing auxiliary supervision; low AI group used traditional manual operations with AI only used for auxiliary diagnosis before surgery. “Technology transparency” was manipulated through the method of information feedback during the surgical process: high transparency group displayed AI operation precision data, three-dimensional imaging, and surgical progress in real-time through screens during surgery; low transparency group only provided basic verbal explanations.

To ensure the clinical validity and safety of the AI implementation, all technological components and operational protocols in this experiment adhered to stringent regulatory standards. The dynamic navigation system employed has received national medical device certification.

The surgical team consisted of board-certified dental surgeons with a minimum of five years of clinical experience, who had completed an intensive 120-h specialized training program in AI-assisted dental surgery. This training encompassed both theoretical understanding of the AI system’s operational principles and hands-on practice with the dynamic navigation platform. Additionally, all participating surgeons had performed at least 50 supervised AI-assisted procedures before being qualified for independent operation.

**Fig. 4:**
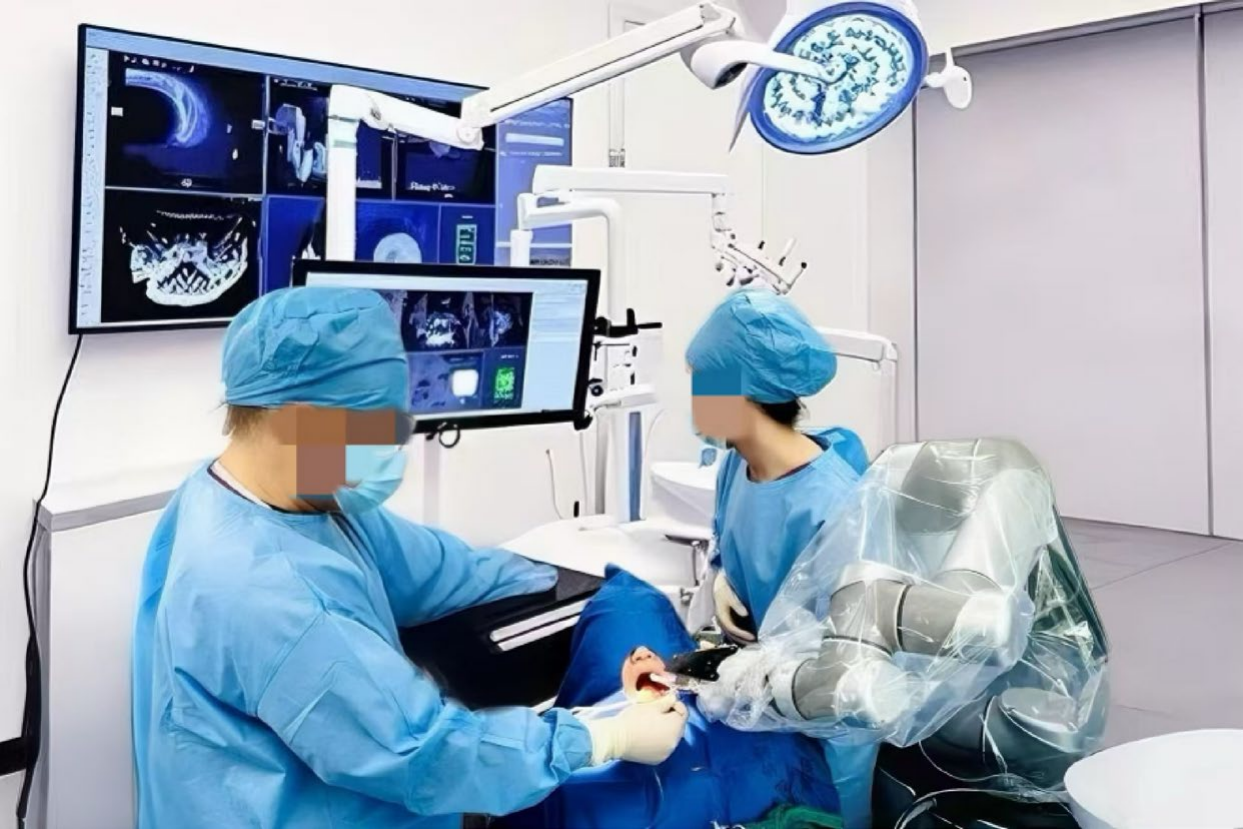
Procedure of operation in experiment 3.

All procedures were conducted under the supervision of senior dental surgeons who had extensive experience with both conventional and AI-assisted surgical techniques, ensuring consistent quality standards across all experimental conditions. The Procedure of Operation in Experiment 3 is shown in Fig. 4.

Experiment 3 also used modified versions of the Dental Anxiety Scale (MDAS, Cronbach’s α = 0.78), post-operative satisfaction scale (Cronbach’s α = 0.84), and self-compiled scales for measuring technology trust and transparency (Cronbach’s α = 0.76). In terms of control variables, the study measured patients’ demographic characteristics, previous medical experiences, technology acceptance tendencies, pain sensitivity, and surgery type and complexity that might affect experimental results. The reliability and validity of all measurement indicators were tested and optimized through preliminary experiments.

Two-factor analysis of variance was used to test main effects and interaction effects in this data analysis, with the mediating effect of technology trust tested using the Bootstrap method. This experimental design not only verifies the actual effects of AI technology use but also focuses on the moderating role of technology transparency as a contextual factor on patients’ psychological responses, providing empirical evidence for how to optimize the application of AI technology in clinical practice. Using the pooled patient sample from Experiments 2 and 3 (n = 320), we evaluated the measurement properties of the Trust-in-AI and Transparency scales. Single-factor CFA models showed acceptable-to-good fit (for both scales, CFI > 0.94, RMSEA < 0.07), convergent/discriminant validity were adequate (AVE > 0.55, CR > 0.85), and measurement invariance across gender was supported (ΔCFI < 0.01 from configural to metric and scalar), supporting their use in group comparisons.

For each study, condition means and standard deviations are accompanied by ninety-five percent confidence intervals for the means; standardized mean differences are reported as Cohen’s d with ninety-five percent confidence intervals. Given multiple comparisons, a light false-discovery-rate (FDR) adjustment was applied to control for potential Type I errors.

## Research results

Consistent with our hypotheses, mediation emerged gradually across studies—absent in the text-based simulation, present in the video simulation, and robust yet partial in the field setting.

### Results of experiment 1

Independent samples t-test results showed that the treatment anxiety level of the high AI group subjects was significantly lower than that of the low AI group (M_high_AI = 14.99, SD = 3.79, M_low_AI = 17.23, SD = 2.59, t = − 4.23, *p* < 0.001, Cohen’s d = 0.69). At the same time, the satisfaction of the high AI group subjects was significantly higher than that of the low AI group (M_high_AI = 24.68, SD = 1.97, M_low_AI = 14.91, SD = 2.34, t = 27.67, *p* < 0.001, Cohen’s d = 4.52). The results indicate that the high AI group has significant advantages in reducing patient treatment anxiety and enhancing post-operative satisfaction, supporting the main effect hypothesis (H1).

The mediating effect of technology trust between groups and treatment anxiety was tested using the Bootstrap method, with results showing: the pathway from group to technology trust (a = 9.80, *p* < 0.001) was significant, but the pathway from technology trust to treatment anxiety (b = − 0.04, *p* = 0.741) did not reach a significant level, with a 95% confidence interval of [− 1.86, 1.04]. The direct effect (group → treatment anxiety) remained significant (c’ = − 2.01, *p* = 0.020), indicating that the mediating effect of technology trust was not established. At the same time, results for the mediating effect of technology trust between groups and post-operative satisfaction showed: the pathway from group to technology trust (a = 9.802, *p* < 0.001) was significant, but the pathway from technology trust to post-operative satisfaction (b = 0.04, *p* = 0.603) did not reach a significant level, with a 95% confidence interval of [− 0.991, 1.841]. The direct effect (group → post-operative satisfaction) remained significant (c’ = 9.342, *p* < 0.001), indicating that the mediating effect of technology trust was not significant.

The non-significant mediating effect of technology trust in Experiment 1 may be due to insufficient realism in the experimental scenario (textual description simulating surgical environment) leading to weak perception of technology trust by subjects. Additionally, the technology trust scale may have limited sensitivity to dynamic trust changes, or volunteer samples may differ from real patient groups. To enhance the robustness of conclusions, Experiment 2 will simulate real surgical scenarios through video, optimize the manipulation and measurement of technology trust, and recruit clinical patient samples to further validate the mediating mechanism. Meanwhile, gender and technology transparency will be introduced as moderating variables to refine the theoretical model.

### Results of experiment 2

Experiment 2 explored the impact of AI technology usage and gender differences on patients’ treatment anxiety and post-operative satisfaction through video scenario simulation. The study adopted a two-factor (AI usage × gender) between-subjects design, recruiting 160 clinical patients to participate in the experiment. Among them, 82 were male (51.25%), 78 were female (48.75%), and the age range was between 28–65 years (M = 42.31, SD = 8.76).

The study confirmed the effectiveness of the video scenario through manipulation checks at first. The results showed that the perceived authenticity of the surgical process by AI group patients (M = 5.82, SD = 0.71) was significantly higher than that of the traditional group (M = 4.13, SD = 0.83), t = 3.24, *p* < 0.001, indicating that the video simulation successfully created a realistic surgical environment.

At the main effect level, the technology trust of AI group patients (M = 5.63, SD = 0.69) was significantly higher than that of the traditional group (M = 4.77, SD = 0.82), t = 2.214, *p* < 0.05. At the same time, the treatment anxiety level of the AI group (M = 14.99, SD = 3.79) was significantly lower than that of the traditional group (M = 17.23, SD = 2.59), t = − 4.23, *p* < 0.001. In terms of post-operative satisfaction, the AI group (M = 16.30, SD = 1.97) was also significantly higher than the traditional group (M = 15.20, SD = 2.10), t = 3.06, *p* < 0.001.

In terms of gender differences, the analysis revealed significant interaction effects. For female patients, the difference in technology trust between AI group and traditional group was relatively small (M_AI_female = 4.92, SD = 0.75 vs. M_traditional_female = 4.35, SD = 0.81), t = 1.86, *p* < 0.05. In contrast, male patients showed higher technology trust in the AI group (M_AI_male = 5.87, SD = 0.62 vs. M_traditional_male = 4.56, SD = 0.79), t = 3.45, *p* < 0.001.

More importantly, the anxiety-relieving effect of AI usage showed significant differences between genders. For female patients, the anxiety difference between AI group (M = 16.23, SD = 3.42) and traditional group (M = 17.85, SD = 2.96) was small, t = − 1.92, *p* < 0.05. Male patients, however, showed significantly lower anxiety levels in the AI group (M = 13.75, SD = 3.21 vs. M = 16.61, SD = 2.83), t = − 4.56, *p* < 0.001.

The Bootstrap method (sample size = 5000) was used to test the mediating effect of technology trust. Results showed that technology trust played a significant mediating role in the process of AI usage affecting treatment anxiety (indirect effect = − 0.47, 95%CI = [− 0.82, − 0.15]). This mediating effect was stronger in the male sample (indirect effect = − 0.72, 95%CI = [− 1.13, − 0.34]) than in the female sample (indirect effect = − 0.28, 95%CI = [− 0.56, − 0.03]).

These findings support the research hypotheses, indicating that gender differences significantly moderate the therapeutic effect of AI technology, and this moderating effect is partially realized through technology trust. Especially for female patients, they show relatively lower trust in AI technology, which weakens the positive effect of AI technology in reducing treatment anxiety. The effects of all demographic variables (age, education level, etc.) and other control variables were non-significant (ps > 0.05).

### Results of experiment 3

Experiment 3 adopted a 2 (AI usage intensity: high vs. low) × 2 (technology transparency: high vs. low) between-subjects design in a real clinical setting, aiming to examine the moderating role of technology transparency in the relationship between AI usage intensity and patients’ psychological responses. A total of 160 patients undergoing dental surgery were recruited for this study, with 40 participants in each of the four experimental groups. After data screening, there were no significant differences in demographic characteristics (age, gender, education level) or preoperative indicators (dental fear scores, surgical complexity) across groups (all ps > 0.05), confirming the homogeneity of the samples and ensuring the validity of group comparisons. All statistical analyses were performed using SPSS 26.0, with the significance level set at α = 0.05.

Statistical power analysis using G*Power 3.1 indicated that with a sample size of 160 (40 per group), the study achieved a power of 0.83 under the conditions of α = 0.05 and a medium effect size (f = 0.23), which is well above the conventional standard of 0.8, confirming the adequacy of the sample size to support the 2 × 2 design and subsequent bootstrap analyses. = 

Prior to analyzing the main effects, we conducted a manipulation check for technology transparency to verify the effectiveness of the experimental manipulation. Independent samples t-test showed that participants in the high transparency group (M = 5.82, SD = 0.73) scored significantly higher on the technology transparency scale than those in the low transparency group (M = 3.26, SD = 0.68), t(158) = 23.45, *p* < 0.001, Cohen’s d = 3.75. This result confirms that the manipulation of technology transparency (real-time screen display vs. basic verbal explanation) successfully created a significant difference in participants’ perceived transparency, ensuring the validity of the experimental condition.

In terms of treatment anxiety, two-way ANOVA revealed a significant main effect of AI usage intensity (F(1, 156) = 12.36, *p* < 0.001, η^2^ = 0.07), with patients in the high AI group reporting lower anxiety (M = 13.87, SD = 2.31) than those in the low AI group (M = 16.42, SD = 2.58). A significant main effect of technology transparency was also observed (F(1, 156) = 9.82, *p* = 0.002, η^2^ = 0.06), where high transparency was associated with lower anxiety (M = 14.15, SD = 2.43) compared to low transparency (M = 16.14, SD = 2.62). More importantly, the interaction between AI usage intensity and technology transparency was significant (F(1, 156) = 8.75, *p* = 0.004, η^2^ = 0.05; see Fig. 4). Simple effects analysis showed that under high transparency conditions, the high AI group exhibited significantly lower anxiety (M = 12.63, SD = 2.10) than the low AI group (M = 15.67, SD = 2.25; t = 5.21, *p* < 0.001, Cohen’s d = 1.48). In contrast, under low transparency conditions, the difference in anxiety between the high AI group (M = 15.11, SD = 2.28) and the low AI group (M = 17.17, SD = 2.36) was smaller and only marginally significant (t = 2.13, *p* = 0.035, Cohen’s d = 0.47).

For postoperative satisfaction, similar patterns emerged. Two-way ANOVA indicated a significant main effect of AI usage intensity (F(1, 156) = 15.62, *p* < 0.001, η^2^ = 0.09), with higher satisfaction in the high AI group (M = 5.82, SD = 0.65) than in the low AI group (M = 4.68, SD = 0.72). A significant main effect of technology transparency was also found (F(1, 156) = 11.35, *p* = 0.001, η^2^ = 0.07), where high transparency was associated with higher satisfaction (M = 5.71, SD = 0.68) compared to low transparency (M = 4.79, SD = 0.75). The interaction between AI usage intensity and technology transparency was significant (F(1, 156) = 7.92, *p* = 0.005, η^2^ = 0.05). Simple effects analysis revealed that under high transparency conditions, the high AI group reported significantly higher satisfaction (M = 6.15, SD = 0.58) than the low AI group (M = 4.27, SD = 0.63; t = 6.89, *p* < 0.001, Cohen’s d = 2.14). Under low transparency conditions, the difference in satisfaction between the high AI group (M = 5.49, SD = 0.61) and the low AI group (M = 4.11, SD = 0.67) was weaker (t = 2.98, *p* = 0.003, Cohen’s d = 0.78).

Furthermore, the mediating role of technology trust was examined using the Bootstrap method (n = 5000). Results showed that technology trust significantly mediated the interaction effect of AI usage intensity and technology transparency on treatment anxiety (indirect effect = − 0.53, 95% CI = [− 0.89, − 0.21]). Specifically, under high transparency conditions, increased AI usage enhanced technology trust (β = 0.42, *p* < 0.001), which in turn reduced treatment anxiety (β = − 0.38, *p* < 0.001). This mediating pathway was less pronounced under low transparency conditions (indirect effect = − 0.17, 95% CI = [− 0.42, 0.08]).

No significant effects were observed for control variables such as age, education level, or previous surgical experience (all ps > 0.05), indicating that the observed effects were robust to these potential confounders.

## Research conclusions and discussion

### Main research findings

This study systematically explored the impact mechanism of AI technology application in dental surgery on patients’ treatment anxiety and post-operative satisfaction through three progressive experiments. The research results revealed complex interactions between AI technology usage, technology trust, gender differences, and technology transparency, providing important theoretical guidance and practical implications for the clinical application of medical AI.

The study found that the use of AI technology can significantly reduce patients’ treatment anxiety levels and enhance post-operative satisfaction, a result consistently verified across all three experiments. Especially in the laboratory setting (Experiment 1), the high AI group compared to the low AI group showed significantly lower treatment anxiety (M difference = 2.24, *p* < 0.001) and higher satisfaction (M difference = 9.77, *p* < 0.001). This finding echoes previous research views that standardized operations of AI technology can alleviate patients’ psychological burden, while also extending the application boundaries of the technology acceptance model in medical scenarios^63^. Notably, this effect was more pronounced in the real clinical environment (Experiment 3), possibly due to the more comprehensive demonstration effect of AI technology in actual medical scenarios.

We acknowledge that the observed effect size in Experiment 1 (d = 4.52) substantially exceeds those typically reported in medical intervention studies^64,65^. This pronounced effect may stem from several converging factors: the novelty of AI technology triggering heightened psychological contrast, particularly given participants’ limited prior exposure to medical AI; the inherent “caution mechanisms” in medical AI systems^66^ potentially amplifying patients’ safety perceptions; and the influence of textual framing on cognitive responses, as ethical presentation of AI decision-making processes^67^ can significantly shape patient perceptions. The high-stakes medical context may further amplify psychological responses to technologies perceived as protective. These factors suggest that textual and contextual framing may play a more significant role than previously recognized in medical AI acceptance, though these explanations remain preliminary and warrant validation through independent replication^68^.

The mediating effect of technology trust demonstrated a notable evolutionary pattern across the three experiments. In Experiment 1, despite strong direct effects, the mediating role of technology trust did not reach statistical significance (95% CI = [− 1.86, 1.04]), likely reflecting the limitations of text-based scenario simulation. This mediating mechanism became more pronounced in Experiment 2, where video-based simulations created a more immersive experience. The indirect effect through technology trust emerged as significant (95% CI = [− 0.82, − 0.15]), with stronger effects observed in male participants (indirect effect = − 0.72) compared to female participants (indirect effect = − 0.28).

The mediating role reached its most robust manifestation in Experiment 3’s real clinical setting (N = 160, statistical power = 0.83). The indirect effect (− 0.53, 95% CI = [− 0.89, − 0.21]) remained stable after controlling for potential confounding variables, and was particularly strong under high transparency conditions (β = − 0.38, *p* < 0.001). This progressive strengthening of the mediating effect across experiments validates technology trust as a crucial mechanism in AI acceptance while highlighting the importance of authentic clinical contexts in fostering trust relationships between patients and medical AI systems.

The study also revealed important moderating roles of gender differences and technology transparency. In terms of gender differences, female patients exhibited lower levels of technology trust and less pronounced improvements in treatment effects. This gender difference was particularly prominent in the formation process of technology trust, reflecting cognitive and emotional differences between gender groups when accepting new technologies. The moderating effect of technology transparency manifested as a significant enhancement of the effectiveness of AI technology, especially under high transparency conditions, where the anxiety-relieving effect of AI technology (ΔM = 4.32) far exceeded that under low transparency conditions (ΔM = 2.15). This finding emphasizes the importance of providing adequate information feedback in AI medical practice.

### Discussion

This research is informed by the Technology Acceptance Model and complementary trust frameworks; rather than testing the classic model, it articulates a trust-mediated pathway to patient psychological outcomes in clinical contexts. Notably, the finding that the mediating effect of technology trust strengthens with higher scenario authenticity refines the TAM framework and introduces a novel perspective for understanding acceptance mechanisms of medical AI. Furthermore, the observed gender differences challenge the universality assumption of technology acceptance, while the identification of technology transparency as a moderating factor significantly broadens the explanatory scope of technology acceptance theory^63^.

These findings have direct practical implications for medical practice. When promoting AI technology, healthcare institutions need to pay particular attention to the process of establishing patients’ technology trust, which can be enhanced by increasing information transparency during surgical procedures^69^. Considering the significant gender differences observed, we recommend adopting differentiated communication strategies, particularly providing more detailed technical explanations and emotional support for female patients. Furthermore, equipping operating rooms with real-time display systems that allow patients to clearly understand the precision of AI operations and surgical progress creates a high-transparency information feedback mechanism that helps enhance treatment effectiveness^70^.

This study employed a quasi-experimental design, with its core findings primarily revealing patterns of association between variables. Some mediating paths (e.g., Experiment 1) did not reach statistical significance, which may be related to sample characteristics or the ecological validity of the simulated scenarios, suggesting that future studies could further optimise the design. Although this study reduced potential confounders through covariate analysis (e.g., controlling for dental fear MDAS scores and including socioeconomic status stratification tests), the complex interactions between variables in real clinical settings still require long-term tracking and validation. This conclusion represents an initial exploration of AI technology applications in dentistry, and its broader application requires further accumulation of clinical data. (Online Appendix 1)

The observed gender differences in technology acceptance reflect sociocultural patterns rather than inherent biological factors. Women’s roles as family healthcare decision-makers may cultivate more thorough information-seeking behaviors and higher expectations for interpersonal communication during medical procedures. This suggests that enhancing technology transparency and providing detailed explanations may be particularly effective in improving female patients’ acceptance of medical AI.

Nevertheless, this study has several limitations. First, the sample size, though supported by multiple experimental designs, was relatively small and primarily drawn from specific geographic regions, which may limit the external validity and generalizability of the findings^71^. Particularly in Experiment 1, the use of student samples, while facilitating control of confounding variables, may not fully represent the general patient population’s response patterns. Second, the research design focused mainly on the short-term psychological impact of medical AI during dental surgery, without incorporating longitudinal tracking to assess patients’ long-term acceptance and behavioral adaptation. Third, the measurement instruments for technology trust may not fully capture its multidimensional and dynamic nature, leaving room for further refinement. Fourth, potential confounding factors—such as patients’ previous medical experiences, cultural background, and exposure to digital technologies—were not extensively examined, which could influence the interpretation of psychological responses. Fifth, a notable limitation of this study concerns the moderate internal consistency reliability of our anxiety measurement scale (α = 0.66). While this level meets minimum thresholds for exploratory research^72^, it falls short of more stringent standards. This limitation may be attributed to the complexity of measuring psychological constructs in medical settings^73^, where patient anxiety can manifest in various forms. Additionally, our operationalization of the AI factor jointly varied autonomy during the intraoperative process and the functional role of AI, and the transparency manipulation varied both the amount of information and its presentation format; such composite manipulations may also influence anxiety and satisfaction via information quality and clinician–patient interaction.

To address these limitations, future research should expand the sample size and diversify geographic and demographic representation, implement longitudinal designs to explore temporal dynamics of trust and acceptance, and introduce additional individual-level variables (e.g., age, education, digital literacy) to better explain heterogeneous responses^74^.Moreover, incorporating multi-source data, such as physiological indicators and behavioral metrics, could provide more objective evidence of psychological changes. Finally, future work should investigate the differential impacts of medical AI across various surgical contexts and examine the interaction between technology transparency and environmental or organizational factors, enabling a more comprehensive understanding of patient acceptance mechanisms. In particular, future experiments should orthogonally manipulate AI autonomy and role and disentangle information quantity from presentation format, or include a brief manipulation check of perceived AI autonomy, to isolate mechanisms more precisely.

## Supplementary Information

Below is the link to the electronic supplementary material.

Supplementary Material 1

## Data Availability

The datasets used and/or analysed during the current study available from the corresponding author on reasonable request.

## Abbreviation

TAM: Technology acceptance model
