# Supplementary material for: An empirical study on the psychological impact of medical AI on patients undergoing dental surgery
Source: Sci Rep. 2025 Dec 3;16:401. doi: 10.1038/s41598-025-29754-0 (PMC12769479; doi:10.1038/s41598-025-29754-0)
Supplement: Supplementary file 1 — Supplementary Material 1 [file 41598_2025_29754_MOESM1_ESM.docx]

## Appendix

## Appendix A：Experiment 1 Questionnaire

### High AI Group Scenario Description:

Please read the following scenario carefully and imagine you are experiencing this medical process:

You visit a dental clinic for treatment. The dentist informs you that the surgery will be performed by the latest generation of intelligent surgical system. This system is equipped with micron-level precision robotic arms and real-time 3D imaging technology, achieving operational precision of 0.1 millimeters, far exceeding the limits of manual operation. During the surgical procedure, the AI system will first conduct a three-dimensional scan and modeling of the entire oral cavity, precisely locating the areas requiring treatment. Subsequently, the system will autonomously complete all operational steps including location planning, anesthetic injection, core treatment, and wound management. The attending physician monitors the entire surgical process in real-time via a control console and can pause or take over the surgery at any time if necessary. The system is equipped with comprehensive safety protection mechanisms that immediately stop and alert if any abnormal situation is detected. Notably, this system has been certified by the National Medical Products Administration and has completed over 10,000 surgeries globally, with surgical outcomes and safety significantly superior to traditional surgical methods.

### Low AI Group Scenario Description:

Please read the following scenario carefully and imagine you are experiencing this medical process:

You visit a dental clinic for treatment. The dentist informs you that the surgery will employ traditional manual operation methods combined with an AI-assisted diagnostic system. This approach combines the dentist's rich clinical experience with AI auxiliary functions and represents the most common standard treatment process currently available. During the surgical procedure, all core operations will be personally performed by the experienced attending physician, including pre-operative examination, treatment planning, anesthetic injection, treatment procedures, and wound management. The AI system provides auxiliary support, primarily including pre-operative image analysis, surgical difficulty assessment, and treatment plan references. This human-machine collaborative model both preserves the physician's professional judgment and rich experience while leveraging AI technology to improve diagnostic accuracy and safety. The clinic has successfully completed thousands of surgeries using this treatment model, maintaining patient satisfaction consistently above 95%. The attending physician has over 10 years of surgical experience in clinical frontline practice and is proficient in various treatment techniques.

### Part One: Informed Consent Statement

Description: This research aims to explore how the application of AI technology in dental surgery affects patient experience. Your responses will be kept strictly confidential and used only for academic research. Participation is entirely voluntary, and you may withdraw at any time. Please read carefully and check "Agree" to continue. □ I agree to participate in this research

### Part Two:

Description: Please select the option that best matches your true feelings (select only one option per question, mark with "√").

| **Questions** | **Relaxed** | **Slightly uneasy** | **Tense** | **Afraid or anxious** | **Very afraid or very anxious** |
| --- | --- | --- | --- | --- | --- |
| 1. If you had to go to the dentist today, how would you feel at home? | □ | □ | □ | □ | □ |
| 2. Imagine you are now in the waiting room waiting to enter the dental clinic, how do you feel? | □ | ☑ | □ | □ | □ |
| 3. Imagine you are sitting in the dental treatment chair, how do you feel when the doctor is preparing to drill your tooth? | □ | □ | □ | □ | □ |
| 4. Imagine you are sitting in the dental treatment chair, how do you feel when the doctor is preparing to clean your teeth? | □ | □ | □ | □ | □ |
| 5. Imagine you are sitting in the dental treatment chair, how do you feel when the doctor is preparing to give you an anesthetic? | □ | □ | □ | □ | □ |

### Part Three: Technology Trust Scale

Description: Please select the option that best matches your view of AI technology (1=strongly disagree, 7=strongly agree).

| **Questions** | 1 | 2 | 3 | 4 | 5 | 6 | 7 |
| --- | --- | --- | --- | --- | --- | --- | --- |
| 1. I believe AI robots can accurately complete dental surgical operations. | □ | □ | □ | □ | □ | □ | □ |
| 2. I worry that AI robots may experience technical failures. (Reverse scoring) | □ | □ | □ | □ | □ | □ | □ |
| 3. The AI operation process is easy for me to understand. | □ | □ | □ | □ | □ | □ | □ |
| 4. Using AI technology makes me feel more at ease. | □ | □ | □ | □ | □ | □ | □ |
| 5. I trust the safety of AI technology in dental surgery. | □ | □ | □ | □ | □ | □ | □ |

### Part Four: Post-operative Satisfaction Scale

Description: Please select the option that best matches your experience (1=very dissatisfied, 7=very satisfied).

| **Questions** | 1 | 2 | 3 | 4 | 5 | 6 | 7 |
| --- | --- | --- | --- | --- | --- | --- | --- |
| 1.I am satisfied with the overall effect of this surgery. | □ | □ | □ | □ | □ | □ | □ |
| 2. The AI operation process makes me feel at ease. | □ | □ | □ | □ | □ | □ | □ |
| 3.Compared to traditional surgery, AI surgery causes less pain. | □ | □ | □ | □ | □ | □ | □ |
| 4. I am willing to choose AI robots for dental surgery again. | □ | □ | □ | □ | □ | □ | □ |
| 5.I am confident about the future application of AI technology. | □ | □ | □ | □ | □ | □ | □ |

### Part Five: Demographic Information

1. Gender: □ Male □ Female □ Other
2. Age: ________ years
3. Age at first dental visit: ________ years
4. Previous dental treatment experience: □ Never received □ 1-2 times □ 3-5 times □ More than 5 times
5. How is your oral health? □ Very healthy (no cavities, gingivitis) □ Occasional problems (gum swelling, pain, etc.) □ Some problems (1-2 cavities and gum swelling) □ Poor (multiple cavities or gum bleeding)
6. Have you ever used AI-assisted medical devices (such as smart wristbands, AI diagnostic tools)? □ Yes □ No
7. How sensitive are you to pain? □ Very insensitive (almost don't feel pain) □ Average □ Very sensitive (even slight pain is difficult to bear)
8. Experimental group (to be filled by researcher): □ High AI group (full AI operation) □ Low AI group (traditional surgery, AI only assists)

## Appendix B：Experiment 2 Questionnaire

### Part One: Informed Consent Statement

Description: This research aims to explore how the application of AI technology in dental surgery affects patient experience. Your responses will be kept strictly confidential, used only for academic research, and no information about you or experimental data will be disclosed. Participation is entirely voluntary, and you may withdraw at any time. Please read carefully and check "Agree" to continue. □ I agree to participate in this research

### Part Two:Screenshots of videos viewed by patients and a description of this technology

The video materials adopted in Experiment 2 centered on the "operation process of dynamic navigation systems in dental implant surgery." These videos aimed to precisely manipulate the independent variable of AI technology usage intensity through standardized dynamic visual presentation, while providing a reliable stimulus carrier for exploring the moderating effect of gender differences on patients' psychological responses. The videos were produced based on real clinical scenarios, strictly adhering to the operational specifications of dental implant surgery, and ensuring the purity of experimental variables through structured design.

As shown in Figure 1, Figure 2, and Figure 3,The total duration of the video is 9 minutes, shot in 4K high-definition quality, with multiple angles including main view and close-up shots to present surgical details. There are no subjective emotional inducement elements throughout the video. The first 1 minute and 30 seconds of the video focus on the preoperative preparation stage, highlighting the preoperative planning function of the dynamic navigation system: in the screen, medical staff operate a 3D oral scanner to scan the alveolar bone of the simulated patient, and the navigation system synchronously generates a real-time 3D reconstruction model. The screen clearly marks the implant site, bone density distribution, and preset implantation path. A voiceover explains simultaneously, "Dynamic navigation technology can achieve precise planning of the implantation path through infrared positioning and computer algorithms, with an error controlled within 0.1mm." This part aims to allow viewers to intuitively understand the core advantage of this technology—relying on AI algorithms for preoperative accurate evaluation, laying the foundation for subsequent surgical operations.

The middle 5 minutes and 45 seconds of the video constitute the core surgical process, which is the key link to distinguish the intensity of AI technology usage between the high-AI group and the low-AI group. In the video for the high-AI group, the screen focuses on the active operation of the dynamic navigation machine: the implant handpiece works in coordination with the navigation positioning device. The AI system dynamically updates the implantation path guide line on the screen by real-time capturing of tiny changes in the oral anatomical structure. When the operation angle deviates from the preset value, the system automatically emits a prompt sound and displays correction suggestions. The voiceover emphasizes, "At this stage, the AI navigation system dominates the control of the implantation path, and the doctor only assists in the operation." Meanwhile, close-up shots show the system's real-time monitoring interface for parameters such as implantation depth and torque, strengthening the perception that "AI technology ensures operational accuracy throughout the process." In contrast, the video for the low-AI group adjusts the expression logic, with more shots showing details of the doctor adjusting the operation based on experience. The voiceover is changed to, "The doctor independently completes the operation with reference to the preoperative planning of the navigation system, and the system only provides reference data," weakening the active decision-making role of AI to form a significant difference in technology usage intensity between the two groups of videos.

The final 2 minutes of the video focus on the postoperative verification stage, mainly presenting the postoperative evaluation function of dynamic navigation technology: the system automatically generates a comparison report between the preoperative planning and the actual implantation path. The screen displays the deviation value through 3D model superposition (all within clinically safe ranges) and synchronously shows the initial stability data of the implant. The voiceover supplements, "Dynamic navigation technology can realize the whole-process data traceability of the operation, providing an objective basis for the evaluation of postoperative effects," further strengthening viewers' perception of the reliability of this technology.

It is worth noting that the surgical environment, instrument models, and medical staff operation specifications in the two groups of videos are completely consistent. The difference in the degree of AI technology participation is only distinguished through voiceover expressions, shot focus distribution, and screen interface display content, so as to exclude the interference of irrelevant variables.

In addition, regarding the video materials used in Experiment 2, we can only provide detailed descriptions and some key screenshots, but cannot disclose the complete video, mainly for the following reasons:

First, the video materials are derived from real clinical surgical scenarios. Although the patient's facial features, personal information, and other details have been desensitized, the complete video may still contain identifiable oral anatomical details or operating room environment characteristics. In accordance with the requirements of the Ethics Committee (approval numbers: fsyy2024022, fsyy2025047), medical images involving patient privacy must be strictly restricted in terms of dissemination scope, and can only be used for internal research analysis. They cannot be submitted as public attachments without special approval, so as to maximize the protection of the rights and interests of the subjects.

Second, the video contains content such as the operation interface of the dynamic navigation system for dental implants and real-time data transmission protocols, which involve the technical patents and trade secrets of the cooperating enterprises. The disclosure of the complete video may lead to the leakage of core technical details, violating the scientific research cooperation confidentiality agreement signed by both parties. Therefore, only key information can be presented through text descriptions and non-confidential screenshots (such as general surgical process images).

Finally, from the perspective of research standardization, the video used in the experiment, as a standardized stimulus material, must maintain strict control over its integrity and originality. If the complete video is made public, it may be re-edited or tampered with by irrelevant personnel, affecting the verification of the reproducibility of the research. Providing reviewed descriptions and screenshots can not only meet the reviewers' needs for understanding the experimental materials but also avoid the above risks, which is in line with the general guidelines for data protection and intellectual property management in medical scientific research.

**The Video Screenshot：**


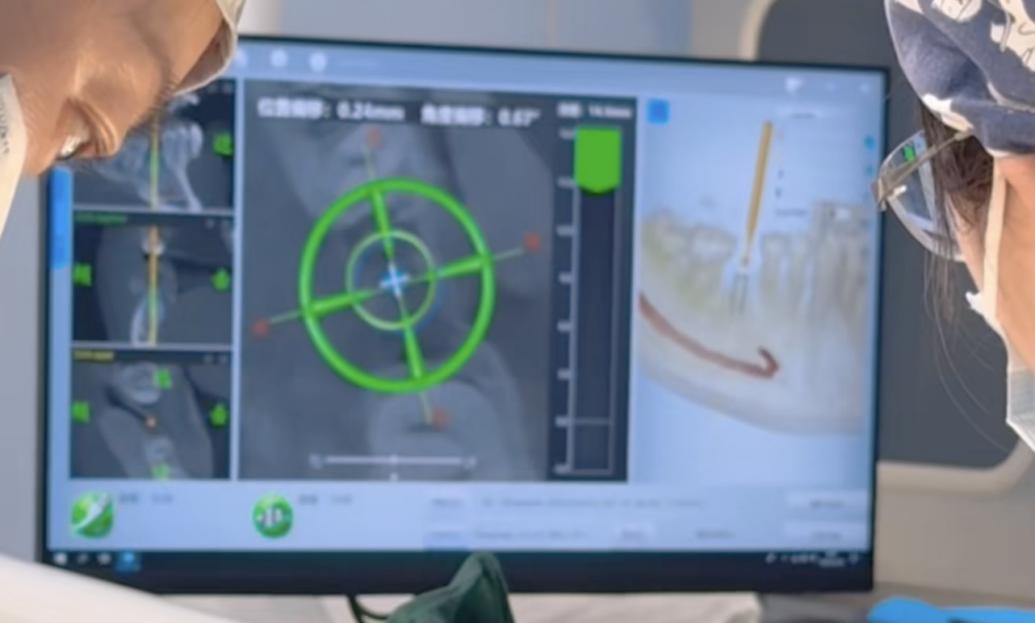


**Figure 1** The doctor operates the dynamic navigation machine for implantation


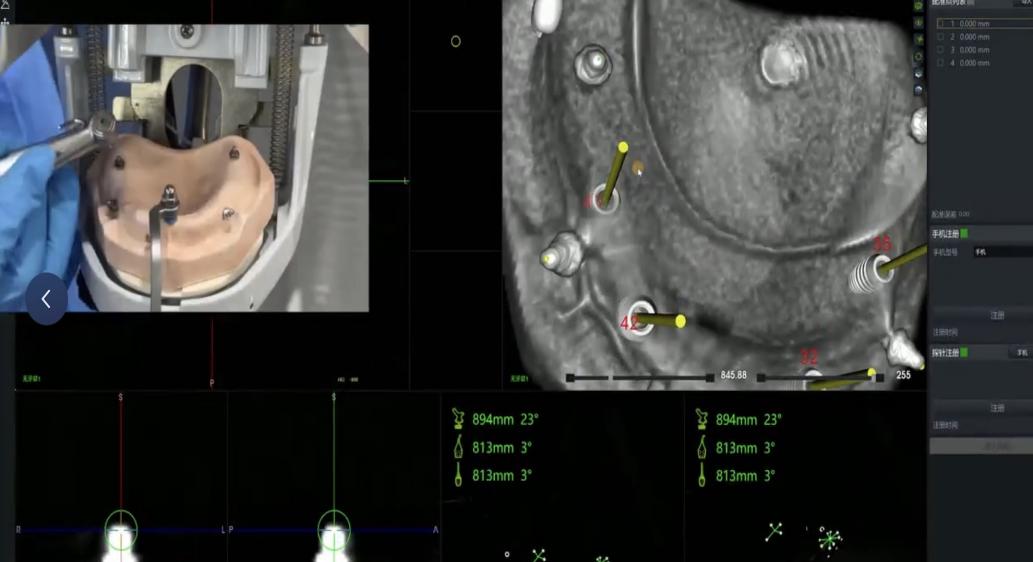


**Figure 2** Dynamic navigation machine interface for planting


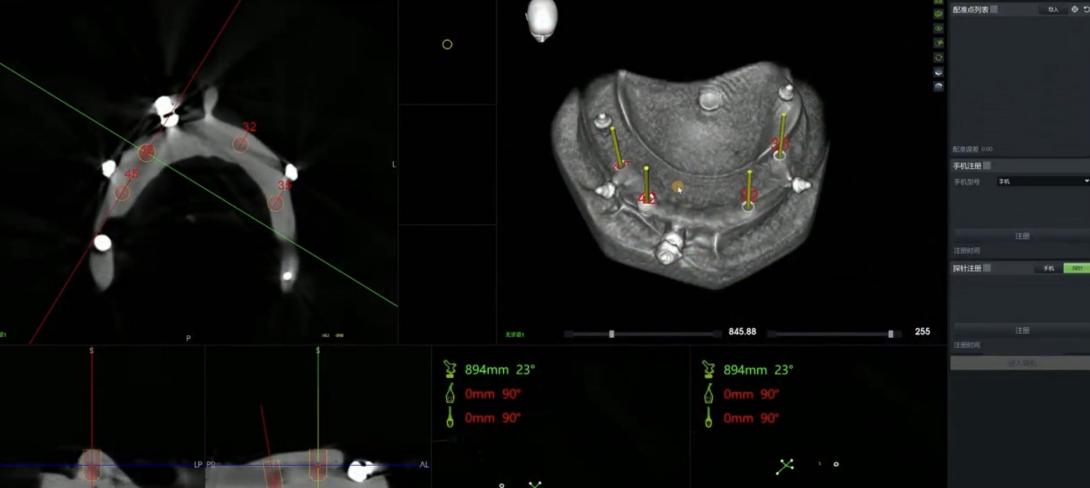


**Figure 3** The interface and actual operation diagram of the dynamic navigation machine for planting

### Part Three: Modified Dental Anxiety Scale (MDAS)

Scoring criteria:

- Relaxed = 1 point
- Slightly uneasy = 2 points
- Tense = 3 points
- Afraid or anxious = 4 points
- Very afraid or very anxious, to the point of feeling physical discomfort = 5 points

Description: Please select the option that best matches your true feelings (select only one option per question, mark with "√").

| **Questions** | **Relaxed** | **Slightly uneasy** | **Tense** | **Afraid or anxious** | **Very afraid or very anxious** |
| --- | --- | --- | --- | --- | --- |
| 1. If you had to go to the dentist today, how would you feel at home? | □ | ☑ | □ | □ | □ |
| 2. Imagine you are now in the waiting room waiting to enter the dental clinic, how do you feel? | □ | □ | □ | □ | □ |
| 3. Imagine you are sitting in the dental treatment chair, how do you feel when the doctor is preparing to drill your tooth? | ☑ | □ | □ | □ | □ |
| 4. Imagine you are sitting in the dental treatment chair, how do you feel when the doctor is preparing to clean your teeth? | □ | □ | □ | □ | □ |
| 5. Imagine you are sitting in the dental treatment chair, how do you feel when the doctor is preparing to give you an anesthetic? | □ | □ | □ | □ | □ |

### Part Four: Technology Trust Scale

Description: Please select the option that best matches your view of AI technology (1=strongly disagree, 7=strongly agree).

| **Questions** | 1 | 2 | 3 | 4 | 5 | 6 | 7 |
| --- | --- | --- | --- | --- | --- | --- | --- |
| 1. I believe AI robots can accurately complete dental surgical operations. | □ | □ | □ | □ | □ | □ | □ |
| 2.I worry that AI robots may experience technical failures. (Reverse scoring) | □ | □ | □ | □ | □ | □ | □ |
| 3.The AI operation process is easy for me to understand. | □ | □ | □ | □ | □ | □ | □ |
| 4.Using AI technology makes me feel more at ease. | □ | □ | □ | □ | □ | □ | □ |
| 5. I trust the safety of AI technology in dental surgery. | □ | □ | □ | □ | □ | □ | □ |

### Part Five: Post-operative Satisfaction Scale

Description: Please select the option that best matches your experience one week after surgery (1=very dissatisfied, 7=very satisfied).

| **Questions** | 1 | 2 | 3 | 4 | 5 | 6 | 7 |
| --- | --- | --- | --- | --- | --- | --- | --- |
| 1. I am satisfied with the overall effect of this surgery. | □ | □ | □ | □ | □ | □ | □ |
| 2. The AI operation process makes me feel at ease. | □ | □ | □ | □ | □ | □ | □ |
| 3. Compared to traditional surgery, AI surgery causes less pain. | □ | □ | □ | □ | □ | □ | □ |
| 4. I am willing to choose AI robots for dental surgery again. | □ | □ | □ | □ | □ | □ | □ |
| 5.I am confident about the future application of AI technology. | □ | □ | □ | □ | □ | □ | □ |

### Part Six: Demographic Information

1. Gender: □ Male □ Female □ Other
2. Age: ________ years
3. Age at first dental visit: ________ years
4. Education level: □ Below high school □ Bachelor's degree/College diploma □ Master's degree and above
5. How is your oral health? □ Very healthy (no cavities, gingivitis) □ Occasional problems (gum swelling, pain, etc.) □ Some problems (1-2 cavities and gum swelling) □ Poor (multiple cavities or gum bleeding)
6. Have you ever used AI-assisted medical devices (such as smart wristbands, AI diagnostic tools)? □ Yes □ No
7. How sensitive are you to pain? □ Very insensitive (almost don't feel pain) □ Average □ Very sensitive (even slight pain is difficult to bear)
8. Experimental group (to be filled by researcher): □ AI video group (receiving surgery led by AI robots) □ Traditional video group (receiving traditional manual surgery)

## Appendix C：Experiment 3 Questionnaire

### Part One: Informed Consent Statement

Description: This research aims to explore how AI technology transparency affects patient experience. Your responses will be kept strictly confidential, used only for academic research, and no information about you or experimental data will be disclosed. Participation is entirely voluntary, and you may withdraw at any time. Please read carefully and check "Agree" to continue. □ I agree to participate in this research

### Part Two: Modified Dental Anxiety Scale (MDAS)

Scoring criteria:

- Relaxed = 1 point
- Slightly uneasy = 2 points
- Tense = 3 points
- Afraid or anxious = 4 points
- Very afraid or very anxious, to the point of feeling physical discomfort = 5 points

Description: Please select the option that best matches your true feelings (select only one option per question, mark with "√").

| **Questions** | **Relaxed** | **Slightly uneasy** | **Tense** | **Afraid or anxious** | **Very afraid or very anxious** |
| --- | --- | --- | --- | --- | --- |
| 1. If you had to go to the dentist today, how would you feel at home? | □ | □ | □ | □ | □ |
| 2. Imagine you are now in the waiting room waiting to enter the dental clinic, how do you feel? | □ | □ | □ | □ | □ |
| 3.Imagine you are sitting in the dental treatment chair, how do you feel when the doctor is preparing to drill your tooth? | □ | □ | □ | □ | □ |
| 4. Imagine you are sitting in the dental treatment chair, how do you feel when the doctor is preparing to clean your teeth? | □ | □ | □ | □ | □ |
| 5. Imagine you are sitting in the dental treatment chair, how do you feel when the doctor is preparing to give you an anesthetic? | □ | □ | □ | □ | □ |

### Part Three: Technology Trust and Transparency Assessment

Description: Please select the option that best matches your experience during the surgical process (1=strongly disagree, 7=strongly agree).

| **Questions** | 1 | 2 | 3 | 4 | 5 | 6 | 7 |
| --- | --- | --- | --- | --- | --- | --- | --- |
| 1. Real-time visualization interface makes me clearer about AI operation steps. | □ | □ | □ | □ | □ | □ | □ |
| 2. I can understand the purpose and effect of each step of AI operation. | □ | □ | □ | □ | □ | □ | □ |
| 3. The doctor's explanation of AI technology makes me feel more at ease. | □ | □ | □ | □ | □ | □ | □ |
| 4.I worry that AI technology lacks transparency. (Reverse scoring) | □ | □ | □ | □ | □ | □ | □ |
| 5. Transparent operation process enhances my sense of trust. | □ | □ | □ | □ | □ | □ | □ |

### Part Four: Post-operative Satisfaction and Revisit Intention

Description: Please select the option that best matches your experience two weeks after surgery (1=very dissatisfied, 7=very satisfied).

| **Questions** | 1 | 2 | 3 | 4 | 5 | 6 | 7 |
| --- | --- | --- | --- | --- | --- | --- | --- |
| 1. I am satisfied with the overall effect of this surgery. | □ | □ | □ | □ | □ | □ | □ |
| 2. The transparent operation process gives me more confidence in AI technology. | □ | □ | □ | □ | □ | □ | □ |
| 3. Compared to traditional surgery, AI surgery has a shorter recovery time.。 | □ | □ | □ | □ | □ | □ | □ |
| 4. I am willing to recommend AI robots for dental surgery to friends and family. | □ | □ | □ | □ | □ | □ | □ |
| 1. In the future, I will prioritize medical institutions that provide transparent feedback. | □ | □ | □ | □ | □ | □ | □ |

### Part Five: Demographic and Surgical Information

1. Gender: □ Male □ Female
2. Age: ________ years
3. Age at first dental visit: ________ years
4. Education level: □ Below high school □ Bachelor's degree/College diploma □ Master's degree and above
5. Surgery type: □ Tooth extraction □ Dental filling □ Dental implant □ Other (please specify: _________)
6. How is your oral health? □ Very healthy (no cavities, gingivitis) □ Occasional problems (gum swelling, pain, etc.) □ Some problems (1-2 cavities and gum swelling) □ Poor (multiple cavities or gum bleeding)
7. Have you ever used AI-assisted medical devices (such as smart wristbands, AI diagnostic tools)? □ Yes □ No
8. How sensitive are you to pain? □ Very insensitive (almost don't feel pain) □ Average □ Very sensitive (even slight pain is difficult to bear)
9. Technology transparency group (to be filled by researcher): □ High transparency group (real-time visualization of operations) □ Low transparency group (no visualization feedback)
10. AI usage intensity (to be filled by researcher): □ High AI group (full AI operation) □ Low AI group (traditional surgery, AI only assists)
